# An Analysis Regarding the Association Between the *ISLR* Gene and Gastric Carcinogenesis

**DOI:** 10.3389/fgene.2020.00620

**Published:** 2020-06-16

**Authors:** Shu Li, Wei Zhao, Manyi Sun

**Affiliations:** ^1^Department of Gastroenterology and Hepatology, Tianjin Medical University General Hospital, Tianjin, China; ^2^General Data Technology Co., Ltd., Tianjin, China; ^3^Department of Gastroenterology, Tianjin Union Medical Center, Tianjin, China

**Keywords:** *ISLR*, expression, methylation, immune cell infiltration, gastric cancer

## Abstract

For datasets of gastric cancer collected by TCGA (The Cancer Genome Atlas) and GEO (Gene Expression Omnibus) repositories, we applied a bioinformatics approach to obtain expression data for the *ISLR* (immunoglobulin superfamily containing leucine-rich repeat) gene, which is highly expressed in gastric cancer tissues and closely associated with clinical prognosis. Although we did not observe an overall association of *ISLR* mutation, high expression or copy number variation with survival, hypomethylation of four methylated sites (assessed by the probes cg05195566, cg17258195, cg09664357, and cg07297039) of *ISLR* was negatively correlated with high expression levels of *ISLR* and was associated with poor clinical prognosis. In addition, we detected a correlation between *ISLR* expression and the infiltration levels of several immune cells, especially CD8^+^ T cells, macrophages and dendritic cells. We also identified a series of genes that were positively and negatively correlated with *ISLR* expression based on the TCGA-STAD, GSE13861, and GSE29272 datasets. Principal component analysis and random forest analysis were employed to further screen for six hub genes, including *ISLR*, *COL1A2*, *CDH11*, *SPARC*, *COL3A1*, and *COL1A1*, which exhibited a good ability to differentiate between tumor and normal samples. GO (Gene Ontology) and KEGG (Kyoto Encyclopedia of Genes and Genomes) pathway and gene set enrichment analysis data also suggested a potential relationship between *ISLR* gene expression and epithelial-mesenchymal transition (EMT). *ISLR* expression was negatively correlated with sensitivity to PX-12 and NSC632839. Taken together, these results show that the *ISLR* gene is involved in gastric carcinogenesis, and the underlying molecular mechanisms may include DNA methylation, EMT, and immune cell infiltration.

## Introduction

The Cancer Genome Atlas (TCGA), a publicly funded project, archives multiple types of genomic data from various types of cancer, including gene expression, mutation, copy number variation (CNV), genome methylation, and clinical data (Cancer Genome Atlas Research Network, 2014; Tomczak et al., 2015; Wang et al., 2016). In addition, GEO (Gene Expression Omnibus) molecular datasets also offer many clinical cancer-related gene expression data (Barrett et al., 2013; Clough and Barrett, 2016). The complicated pathogenesis of gastric cancer involves multiple clinical prognosis-associated oncogenes. Previously, based on the datasets of gastric cancer within TCGA and GEO, we identified the *ISLR* (immunoglobulin superfamily containing leucine-rich repeat) gene by means of principal component analysis (PCA) and random forest analysis (data not shown), which showed a high expression level in gastric cancer tissues and was closely linked to clinical prognosis. The present study attempted to investigate the possible oncogenic roles of the *ISLR* gene in the pathogenesis and prognosis of gastric cancer.

The human *ISLR* gene is situated on human chromosome 15q23-q24 (Nagasawa et al., 1997). The human ISLR protein, a member of the Ig superfamily, contains a leucine-rich repeat (LRR) with conserved flanking sequences and a C2-type immunoglobulin (Ig)-like domain (Nagasawa et al., 1997). The ISLR protein has been reported to be involved in some biological events, such as cell replicative senescence of human dermal fibroblasts (Yoon et al., 2004), embryo development (Homma et al., 2009), and Gaucher disease (Lugowska et al., 2019). However, no study has mainly investigated the potential functional relationship between the *ISLR* gene and cancer events thus far.

In the current study, we elucidated the underlying molecular mechanisms of the *ISLR* gene in gastric carcinogenesis from the perspectives of genetic mutation, copy number variation, DNA methylation, immune cell infiltration, expression correlation, pathway enrichment and drug sensitivity for the first time.

## Materials and Methods

### Expression Analysis

We first investigated the expression level of the *ISLR* gene between gastric cancer and negative controls samples within the TCGA-STAD (The Cancer Genome Atlas stomach adenocarcinoma) cohort and the GTEx (Genotype Tissue Expression) database using the online tool GEPIA2^1^ (Tang et al., 2019). A log2 (FC) (fold change) cutoff = 1, a *P-*value cutoff = 0.01, and a jitter size = 4 were set. Log_2_ [TPM (transcripts per million) + 1] values were used for log-scale. Gene expression data were visualized by the “boxplot” function of the R language (for the cancer and control samples) or the “vioplot” R package [for the pathological stage (stages I, II, III, and IV)]. Then, we obtained the expression dataset of “Chen et al. (2003),” which contains a total of 11 diffuse gastric adenocarcinoma and 24 normal control samples, by means of Oncomine^2^. The log2 (median-centered intensity) data were visualized by GraphPad Prism software, version 5.01 (San Diego).

Furthermore, we utilized the “GEOquery” R package to obtain the available expression and group datasets in GSE13861 and GSE29272. The difference in expression of the *ISLR* gene between gastric cancer cases and normal controls was analyzed by the t.test function of the compare_means and visualized by the ggviolin function of the “ggpubr” R package. We then used the wilcox.test function of the compare_means with the setting of “paired = TRUE” to analyze the difference in expression between the gastric tumor tissues and adjacent normal tissues and displayed the results using the ggdotchart function of the “ggpubr” R package. R language software [R-3.6.1, 64-bit]^3^ was used.

### Survival Curve Analysis

We conducted OS (overall survival) and DFS (disease-free survival) analyses of gastric cancer cases in the TCGA-STAD cohort according to the expression status of the *ISLR* gene through GEPIA2. A group cutoff of “quartile” was set, and the Kaplan–Meier curve was plotted. We also pooled the gastric cancer cases in the GSE14210, GSE15459, GSE22377, GSE29272, GSE51105, and GSE62254 datasets for the OS, FP (first progression), and PPS (post progression survival) analyses using the Kaplan–Meier plotter tool (Szasz et al., 2016). The automatically selected best cutoff was used. We considered clinical factors including sex (female or male), pathologic stage (stages 1∼4, T2∼4, N0∼3, M0∼1), HER2 status (negative or positive), Lauren classification (intestinal, diffuse, or mixed), differentiation (poor, moderate, or well), and treatment (surgery alone, 5-Fu-based adjuvant or other adjuvant). Furthermore, we employed the Coxph (Cox proportional hazard) model to determine the correlation between *ISLR* expression and the clinical prognosis of gastric cancer cases in TCGA-STAD through the web-based tool TIMER (Tumor Immune Estimation Resource) (Li et al., 2016, 2017). Clinical factors, including age, sex, race, stage, and tumor purity, were included in the Coxph model.

### Genetic Alteration Analysis

The alteration frequency of the *ISLR* gene in several studies of gastric cancer, including the TCGA pub (2014), PanCan 2018 (Ellrott et al., 2018; Gao et al., 2018; Hoadley et al., 2018; Liu J. et al., 2018; Sanchez-Vega et al., 2018; Taylor et al., 2018; Bhandari et al., 2019), TCGA cohort, Pfizer and UHK (Wang et al., 2014), UHK (Wang et al., 2011), and U Tokyo (Kakiuchi et al., 2014) studies, was analyzed via the cBioPortal database^4^. We provided data of genomic alteration type, mutation site profile, OS and D/PFS (disease/progression-free survival) analyses. In addition, we generated a MEXPRESS plot (Koch et al., 2015, 2019) to analyze the CNV types of the *ISLR* gene. The correlation between CNV and the expression level of *ISLR* was also analyzed by Pearson’s test. The overall survival analysis according to the CNV status of the *ISLR* gene (masked CNV ≥ or < −0.019) was performed through UCSC Xena^5^. The log-rank test was performed.

### DNA Methylation Analysis

We analyzed the methylation status of *ISLR* DNA in the gastric cases in the TCGA-STAD cohort through MEXPRESS (Koch et al., 2015, 2019). Pearson’s test was used to determine the correlation between methylation and the expression level of the *ISLR* gene. We determined correlation coefficients (R) and Benjamini–Hochberg-adjusted *P*-values regarding different methylation probes, such as cg05195566, cg15480336, cg02077702, and cg16926502. The waterfall plot of the methylation level of *the ISLR* gene and Kaplan–Meir plots of the relationship between *ISLR* DNA hypermethylation/hypomethylation and cancer survival were generated with the MethSurv tool (Modhukur et al., 2018).

### Immune Cell Infiltration Analysis

We used GEPIA2 to perform pairwise gene correlation analysis between *ISLR* expression and the signatures of the following immune cells: macrophages, TAMs (tumor-associated macrophages), dendritic cells, monocytes, NK (natural killer) cells; mast cells, neutrophils, eosinophils, basophils, B cells, Th1 cells, Th2 cells, Th17 cells, CD8^+^ T cells, Tfh (follicular helper T) cells, resting Treg cells, effector Treg cells, and exhausted T cells. Then, based on a TIMER2 approach, we calculated immune infiltration estimations for TCGA-STAD samples with the TIMER, CIBERSORT, CIBERSORT-ABS, QUANTISEQ, MCPCOUNTER, XCELL, and EPIC algorithms. A heatmap with the purity-adjusted Spearman’s rho value was obtained by the “pheatmap” R package. Specific scatter plots were provided. In addition, the correlation between *ISLR* SNVs and the level of infiltrating immune cells, including dendritic cells, neutrophils, CD8^+^ T cells, CD4^+^ T cells, B cells, and macrophages, was also investigated by the TIMER tool.

### *ISLR*-Correlated Gene Cluster Analysis

We utilized the “TCGAbiolinks” R package to download the gene expression and clinical information data of TCGA-STAD cohorts from the TCGA database. Log_2_ [FPKM (Fragments per Kilobase Million) + 1] values were used for log-scale. The 25/75% quartile cutoff of *ISLR* expression in three datasets, including TCGA-STAD, GSE13861, and GSE29272, was used to define high and low groups of *ISLR* expression. We then analyzed the *ISLR*-correlated genes through the “limma” R package. The positively or negatively correlated significant genes were visualized by the “ggplot2” R package. The “VennDiagram” R package was used to identify the common genes among TCGA-STAD, GSE13861, and GSE29272. Furthermore, the “clusterProfiler” and “pathview” R packages were used for the GO (Gene Ontology) and KEGG (Kyoto Encyclopedia of Genes and Genomes) enrichment analyses. The data were visualized by the functions cnetplot and dotplot. The GOCircle and chord plots using extracellular matrix-associated terms were visualized by the “GOplot” R package.

In addition, we performed *ISLR*-correlated GSEA (gene set enrichment analysis) and pathway activation/inhibition analyses through a LinkedOmics approach (Vasaikar et al., 2018). The following cutoffs were used: simulations = 500, minimum number of genes = 3, and rank criteria = FDR (false discovery rate). The pathway activity module presents the difference in *ISLR* expression between pathway activity groups (activation and inhibition) defined by pathway scores. The pathway activity module presents the difference in gene expression between pathway activity groups (activation and inhibition) defined by pathway scores.

### Principal Component Analysis

Based on the above common differentially expressed genes, we used the prcomp function for principal component analysis (PCA) to classify the normal and tumor sections in the TCGA-STAD, GSE13861, and GSE29272 datasets. A scree plot was obtained by the plot function, and a three-dimensional map [principal component 1 (PC1), PC2, and PC3] was drawn using the “scatterplot3d” package.

After using the “VennDiagram” R package, common hub genes among TCGA-STAD, GSE13861, and GSE29272 were identified. Then, the cor function and “corrplot” R package were used for the Spearman correlation analysis of these hub genes. The scatter plots were then obtained by the “ggpubr” R package. The “factoextra” R package was utilized to show the principal component weight and to generate two-dimensional contribution maps of common hub genes.

### Random Forest Analysis

Based on the above hub genes, we used the “randomForest” package (ntree = 500) to perform random forest modeling. The MDSplot function was used to obtain a multidimensional scale. The mean decrease accuracy and mean decrease Gini values were calculated by the ggdotchart function in the “ggpubr” package. Using the “pROC” package, the receiver operating characteristic (ROC) curve was plotted, and the area under the ROC curve (AUC) value was calculated.

### Drug Sensitivity Analysis

The correlation between *ISLR* and sensitivity to small molecules and/or drugs was investigated using the GSCALite tool (Liu C. J. et al., 2018). Drug sensitivity and gene expression profiling data of cancer cell lines in the Cancer Therapeutics Response Portal (CTRP) were integrated for investigation (Rees et al., 2016; Liu C. J. et al., 2018). The correlation of *ISLR* gene expression with the small molecule/drug sensitivity (half-inhibitory concentration, IC50) was determined through a Spearman correlation analysis.

## Results

### Expression Analysis Data

**FIGURE 1:**
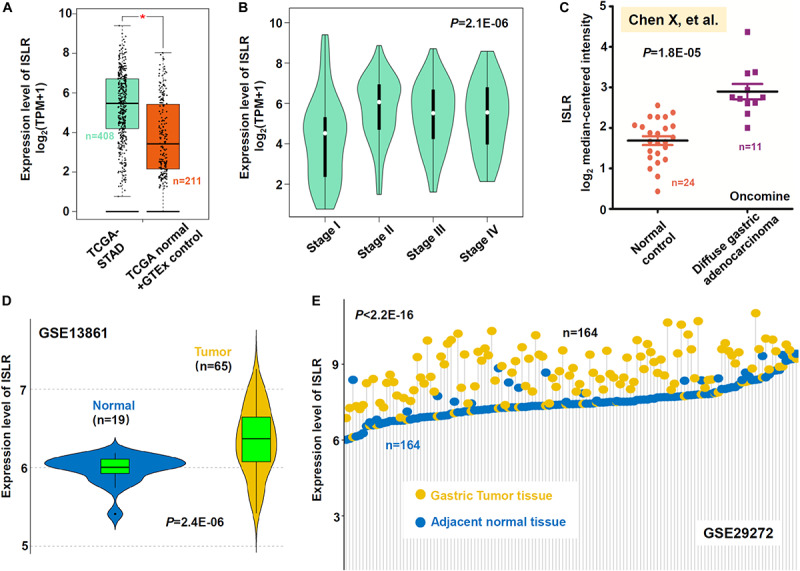
Expression analysis of the *ISLR* gene between gastric cancer and negative control samples. **(A)** Expression level of *ISLR* between gastric cancer tissues in the TCGA-STAD cohort (*n* = 408) and control tissues (*n* = 211). Normal tissues in both the TCGA and GTEx databases were included as negative controls. *P < 0.05. **(B)** The expression level of *ISLR* among different pathologic stages (stage I, II, III, and IV) was also analyzed through GEPIA2. We also compared the expression level of *ISLR* between diffuse gastric adenocarcinoma cases and normal controls from the studies of “Chen et al. (2003)” through the Oncomine database **(C)**. The differences in *ISLR* gene expression between gastric cancer cases and normal controls in GSE13861 **(D)** and GSE29272 **(E)** were analyzed as well. Violin plots were used for GSE13861. Adjacent normal tissues were used for GSE29272, and the data were visualized by a dot plot.

First, the difference in *ISLR* gene expression between gastric cancer tissues and negative control tissues was measured. A total of 408 gastric cancer tissue samples in the TCGA-STAD cohort were included, and the adjacent tissues within TCGA-STAD and normal tissues in the GTEx database were included as negative controls (*n* = 211). As shown in Figure 1A, there was high expression of *ISLR* in the gastric cancer tumor samples (^∗^*P* < 0.05) compared with the controls. We further analyzed the difference in *ISLR* gene expression among different pathological stages of gastric cancer cases in the TCGA-STAD cohort and identified a positive correlation (Figure 1B, *P* = 2.1E-06). Then, based on the dataset reported by Chen et al. (2003), we observed that the expression level of the *ISLR* gene in 11 diffuse gastric adenocarcinoma cases was higher than that in 24 normal controls (Figure 1C, *P* = 1.8E-05). A similar expression difference between tumor and normal samples was detected in the GSE13861 dataset (Figure 1D, *P* = 2.4E-06). Moreover, we observed an obvious high expression level of *ISLR* in 164 gastric tumor tissues compared with 164 adjacent normal tissues within the GSE29272 dataset (Figure 1E, *P* < 2.2E-16). Collectively, these results indicated that the expression level of the *ISLR* gene in gastric cancer cases was higher than that in negative controls, which suggests the potential role of the *ISLR* gene in the etiology of gastric cancer.

### Survival Curve Analysis Data

**FIGURE 2:**
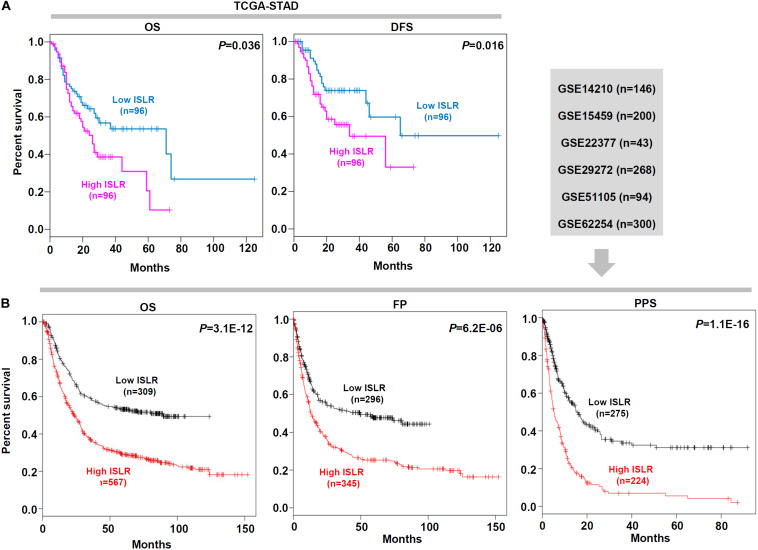
Survival curve analysis of the *ISLR* gene for gastric cancer cases. **(A)** Overall survival (OS) and disease-free survival (DFS) analyses according to the expression level of the *ISLR* gene were performed using gastric cancer cases in the TCGA-STAD cohort. **(B)** Based on the data of gastric cancer cases in GSE14210, GSE15459, GSE22377, GSE29272, GSE51105, and GSE62254, we also performed OS, first progression (FP), and post progression survival (PPS) analyses through Kaplan–Meier plotter.

Next, we explored the correlation between *ISLR* expression patterns and clinical prognosis for gastric cancer cases in the TCGA-STAD cohort. As shown in Figure 2A, we observed lower rates of overall survival (*P* = 0.036) and disease-free survival (*P* = 0.016) in the high *ISLR* expression group than in the low *ISLR* expression group. We also pooled a total of six GSEA datasets for the clinical prognosis analyses. As shown in Figure 2B, there were lower overall survival (*P* = 3.1E-12), first progression (*P* = 6.2E-06), and post progression survival (*P* = 1.1E-16) rates in the *ISLR* high expression group than in the low expression group. Additionally, we fully considered the effect of different clinical factors (e.g., sex, pathologic stage, HER2 status, Lauren classification, differentiation and treatment) during the above analyses. Survival curve analyses were carried out when grouping the samples by the different clinical factors. As shown in Table 1, there was a relationship between high *ISLR* expression and poor overall survival (hazard ratio, HR > 1, *P* < 0.05) in most subgroups but not in the subgroups with poor (*P* = 0.21) or moderate (*P* = 0.086) differentiation, or stage 1 disease (*P* = 0.065). Surprisingly, for the 158 gastric cancer cases treated with 5-Fu-based adjuvant therapy, a high level of *ISLR* expression was linked to a better clinical prognosis than a low level of *ISLR* expression (Table 1, HR = 0.63, *P* = 0.013), indicating a possible connection of *ISLR* expression with drug sensitivity. We observed similar results in the correlation analysis of *ISLR* expression and first progression and post-progression survival (Supplementary Tables S1, S2). Moreover, we included the factors of tumor purity, age, sex, race, clinical stage, and *ISLR* expression in a Cox proportional hazard model and obtained a statistical correlation between high *ISLR* expression and poor clinical prognosis (Table 2, *P* = 0.018). These findings offer evidence regarding the relationship between *ISLR* expression and clinical outcomes. This led us to perform a more in-depth molecular mechanism study.

**TABLE 1:** Correlation of ISLR expression and the overall survival of gastric cancer patients in the GEO cohort (Kaplan***–***Meier plotter).

Factor	Group	Sample size	HR	95% CI	*logRank_P*
Gender	Female	244	2.4	1.57–3.68	**3.2E-05**
	Male	567	2.02	1.58–2.59	**1.2E-08**
Stage	Stage 1	69	2.7	0.9–8.07	0.065
	Stage 2	145	2.04	1.11–3.74	**0.019**
	Stage 3	319	2.72	1.84–4.03	**1.9E-07**
	Stage 4	152	1.82	1.22–2.71	**0.0029**
Stage T	T2	253	1.7	1.12–2.6	**0.013**
	T3	208	1.93	1.33–2.95	**6.0E-04**
	T4	39	2.66	1.06–6.68	**0.032**
Stage N	N0	76	2.95	1.13–7.69	**0.021**
	N1	232	2.74	1.78–4.21	**1.8E-06**
	N2	129	3.15	1.88–5.27	**4.9E-06**
	N3	76	2.08	1.18–3.67	**0.0097**
	N1 + 2 + 3	437	2.22	1.69–2.90	**2.9E-09**
Stage M	M0	459	2.67	1.44–4.96	**0.0012**
	M1	58	2.12	1.59–2.82	**1.6E-07**
HER2	Negative	641	2.09	1.61–2.72	**1.3E-08**
	Positive	425	1.74	1.26–2.39	**0.00058**
Lauren classification	Intestinal	336	2.85	2.00–4.08	**1.7E-09**
	Diffuse	248	1.94	1.36–2.77	**0.00018**
	Mixed	33	3.31	1.17–9.42	**0.018**
Differentiation	Poor	166	1.31	0.86–2.01	0.21
	Moderate	67	1.75	0.92–3.34	0.086
	Well	32	5.97	2.25–15.85	**6.1E-05**
Treatment	Surgery alone	393	1.59	1.19–2.13	**0.0017**
	5-Fu based adjuvant	158	0.63	0.44–0.91	**0.013**
	Other adjuvant	80	3.13	1.30–7.52	**0.0072**

*HR, hazard ratio; CI, confidence interval; HER2, Erb-B2 Receptor Tyrosine Kinase 2. Bold values mean *P* < 0.05.*

**TABLE 2:** Correlation of ISLR expression and the clinical prognosis of gastric cancer patients in the TCGA-STAD cohort (Cox proportional hazard model).

Factor	HR	95% CI_up	95% CI_down	*Cox_P*
*ISLR*	1.161	1.026	1.314	**0.018**
Purity	0.638	0.304	1.338	0.234
Age	1.032	1.011	1.052	**0.002**
Gender (male)	1.133	0.754	1.702	0.549
Race (Black)	1.619	0.657	3.992	0.295
Race (White)	1.109	0.681	1.806	0.679
Clinical stage2	1.471	0.679	3.188	0.328
Clinical stage3	2.428	1.190	4.954	**0.015**
Clinical stage4	3.813	1.422	10.223	**0.008**

*HR, hazard ratio; CI, confidence interval. Bold values mean *P* < 0.05.*

### Genetic Alteration Analysis Data

**FIGURE 3:**
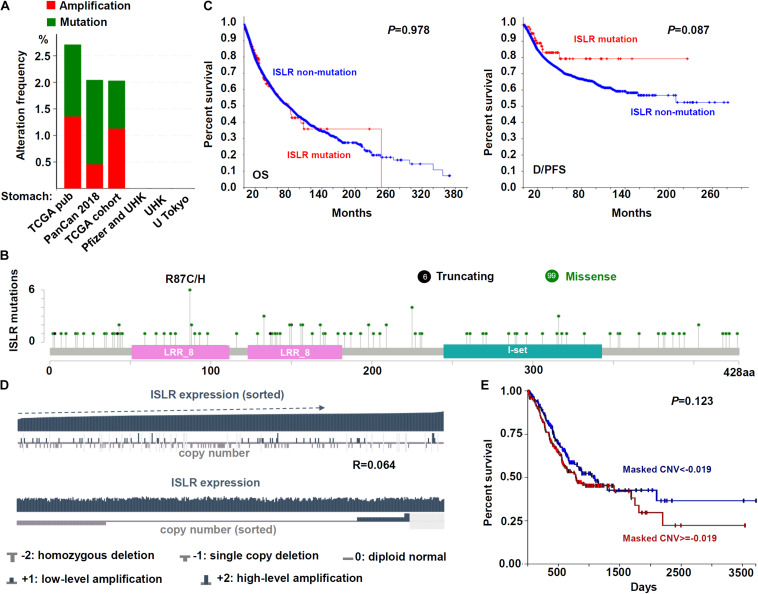
Mutation and CNV analyses of the *ISLR* gene. **(A)** The alteration frequency of the *ISLR* gene for the cases in several studies as analyzed through cBioPortal. **(B)** The mutation site profile of the *ISLR* gene is shown. **(C)** OS and D/PFS analyses according to the mutation status of the *ISLR* gene were performed. **(D)** Correlation between copy number variation and expression of *ISLR*. **(E)** OS analysis according to the CNV status of the *ISLR* gene was performed.

We attempted to study the potential mechanism of the *ISLR* gene in the pathogenesis of gastric cancer in terms of gene mutation and copy number variation. As shown in Figure 3A, we detected the mutation frequency in six groups of gastric cancer cases through the cBioPortal database. There was a low mutation rate (∼2%) of *ISLR* in the cases in the TCGA-STAD, TCGA pub (2014), and PanCan 2018 (Ellrott et al., 2018; Gao et al., 2018; Hoadley et al., 2018; Liu J. et al., 2018; Sanchez-Vega et al., 2018; Taylor et al., 2018; Bhandari et al., 2019) cohorts and no mutation in gastric cancer cases in the Pfizer and UHK (Wang et al., 2014), UHK (Wang et al., 2011), and U Tokyo (Kakiuchi et al., 2014) cohorts. The type and location of specific mutations, with the most frequent missense mutation being R87C/H (*n* = 6), are shown in Figure 3B. Additionally, we did not observe a statistically significant correlation between *ISLR* gene mutation and the OS rate (Figure 3C, *P* = 0.978) or the D/PFS rate (Figure 3C, *P* = 0.087).

Next, we investigated the CNV status of the *ISLR* gene. As shown in Figure 3D, the *ISLR* gene mainly exhibited two kinds of CNVs, namely, single copy deletion and low-level amplification. However, there was no statistically significant association between *ISLR* CNV and gene expression (Figure 3D, *R* = 0.064) or the overall survival rate of gastric cancer cases (Figure 3E, *P* = 0.123). These results suggested that *ISLR* gene mutation and copy number variation may not affect gastric tumorigenesis.

### DNA Methylation Analysis Data

**FIGURE 4:**
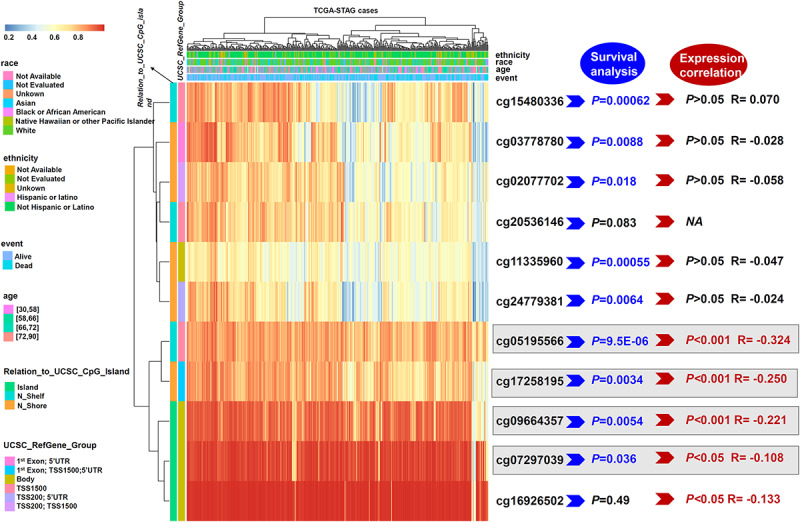
Waterfall plot and analysis of potential methylation probes targeting the *ISLR* gene. A waterfall plot of the methylation level of the *ISLR* gene is provided. The correlations between *ISLR* methylation or expression level and survival rate were also analyzed. NA, not available.

Next, we aimed to investigate whether the *ISLR* gene was closely linked to *ISLR* DNA methylation. Based on methylation data from TCGA-STAD, we observed that the methylation values from four methylation probes, cg05195566, cg17258195, cg09664357, and cg07297039, were negatively correlated with the expression level of the *ISLR* gene (Figure 4, *P* < 0.05). Supplementary Figure S1 presents the specific information of methylation probe sites and the correlation results of *ISLR* gene expression with methylation level. Additionally, some methylation probes showed a correlation between *ISLR* hypomethylation and poor overall survival in gastric cancer (Figure 4 and Supplementary Figure S2, cg05195566, *P* = 9.5E-06; cg17258195, *P* = 0.0034; cg09664357, *P* = 0.0054; cg07297039, *P* = 0.036).

### Immune Cell Infiltration Analysis Data

**FIGURE 5:**
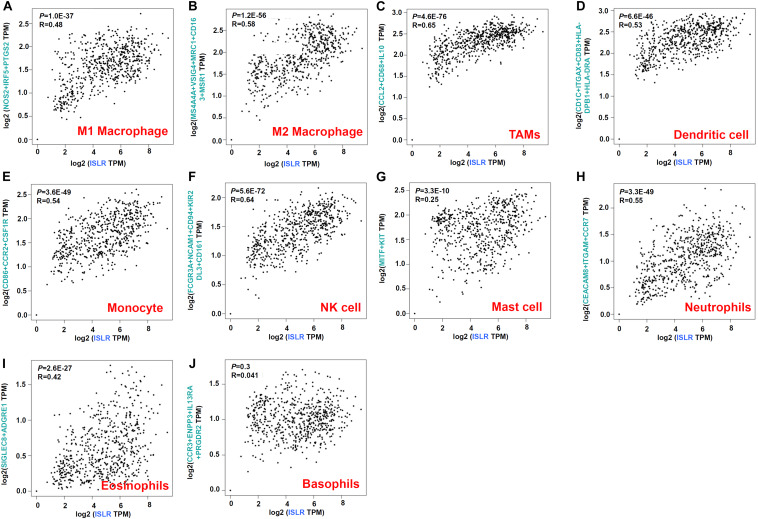
Correlation between *ISLR* expression and markers of immune cells as analyzed through GEPIA2. **(A)** M1 macrophages; **(B)** M2 macrophages; **(C)** TAMs; **(D)** dendritic cells; **(E)** monocytes; **(F)** NK cells; **(G)** mast cells; **(H)** neutrophils; **(I)** eosinophils; **(J)** basophils.

Herein, we sought to explore possible molecular mechanisms through immune cell infiltration during the etiology of gastric cancer. First, through GEPIA2, we analyzed the association between *ISLR* gene expression and immune cell infiltration status. As shown in Figure 5, we observed a positive correlation between *ISLR* expression and the marker genes of M1 macrophages (*R* = 0.48, *P* = 1.0E-37), M2 macrophages (*R* = 0.58, *P* = 1.2E-56), TAMs (*R* = 0.65, *P* = 4.6E-76), dendritic cells (*R* = 0.53, *P* = 6.6E-46), monocytes (*R* = 0.54, *P* = 3.6E-49), NK cells (*R* = 0.64, *P* = 5.6E-72), mast cells (*R* = 0.25, *P* = 3.3E-10), neutrophils (*R* = 0.55, *P* = 3.3E-49), and eosinophils (*R* = 0.42, *P* = 2.6E-27) but not between *ISLR* expression and basophils (*R* = 0.041, *P* = 0.3). We observed similar results for the different types of T and B cells, such as Tfh cells (*R* = 0.56, *P* = 2.7E-52) (*R* = 0.6, *P* = 4.0E-61), and exhausted T cells (Supplementary Figure S3).

**FIGURE 6:**
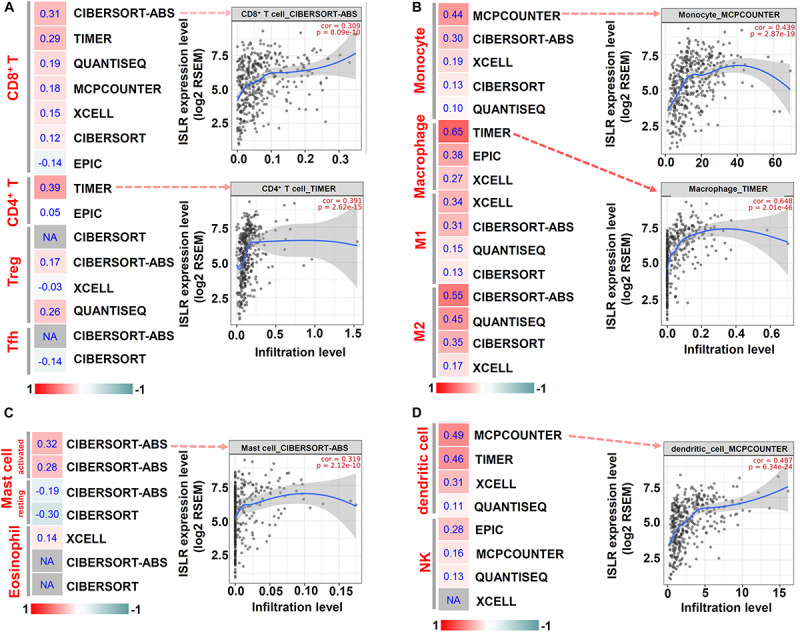
Correlation between *ISLR* expression and the infiltration level of immune cells. The TIMER, CIBERSORT, CIBERSORT.ABS, QUANTISEQ, MCPCOUNTER, XCELL, and EPIC algorithms were applied for the immune infiltration estimations of CD8^+^ T cells, CD4^+^ T cells, Treg cells, and Tfh cells **(A)**; Monocytes and macrophages (M1, M2 type) **(B)**; mast cells and eosinophils **(C)**; and dendritic cells and NK cells **(D)**. A heatmap with the purity-adjusted Spearman’s rho value and specific scatter plots are provided as examples. “NA” means a lack of an association between *ISLR* expression and the infiltration level of immune cells.

Then, we utilized the TIMER, CIBERSORT, CIBERSORT-ABS, QUANTISEQ, MCPCOUNTER, and EPIC algorithms for further immune infiltration estimations. As shown in Figure 6, we observed a relatively obvious correlation between *ISLR* expression and the immune infiltration levels of CD8^+^ T cells, monocytes, macrophages (especially the M2 type), activated mast cells and dendritic cells when adjusted by tumor purity.

Additionally, we detected the correlation between *ISLR* CNV and the overall infiltration level of immune cells (Supplementary Figure S4). The copy deletion type of *ISLR* CNV was correlated with the infiltration level of dendritic cells, neutrophils, CD8^+^ T cells, CD4^+^ T cells, B cells, and macrophages (all *P* < 0.05), while the low-level amplification CNV was only associated with the infiltration level of dendritic cells (*P* < 0.001), neutrophils (*P* < 0.01), and CD8^+^ T cells (*P* < 0.001).

### Cluster Analysis Data

**FIGURE 7:**
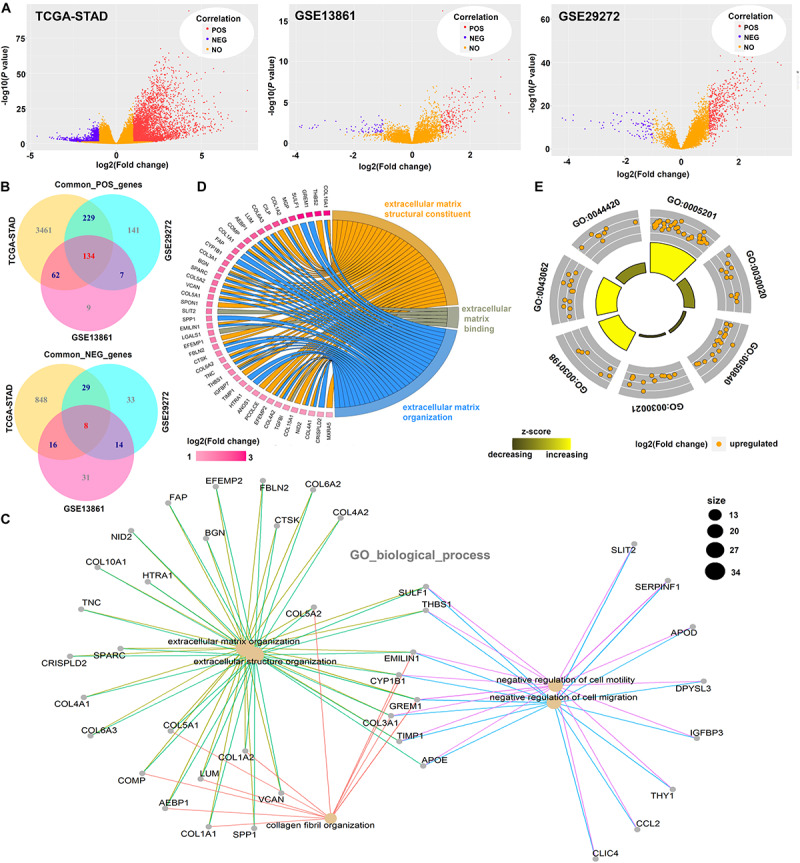
Cluster analysis of significant *ISLR*-correlated genes. **(A)** After expression difference analyses between the ISLR-high and -low expression groups were performed with the “limma” R package on the TCGA-STAD, GSE13861, and GSE29272 datasets, volcano plots were constructed. POS, genes positively correlated with *ISLR*; NEG, genes negatively correlated with *ISLR*; NO, genes not correlated with *ISLR*. **(B)** The “VennDiagram” R package was used for the common genes positively (POS) and negatively (NEG) correlated with ISLR. **(C)** The “clusterProfiler” R package was used for the GO_biological_process enrichment analysis. The GOCircle **(D)** and chord plots **(E)** using extracellular matrix-associated terms were visualized by the “GOplot” R package.

Based on the “limma” R package, we obtained genes positively or negatively correlated with *ISLR* among three datasets: TCGA-STAD, GSE13861, and GSE29272 (Figure 7A). Then, we performed intersection analysis and identified 134 common positively correlated genes and 8 common negatively correlated genes (Figure 7B). Then, we performed GO enrichment analyses. We observed extracellular matrix-associated terms, such as extracellular structure organization and extracellular matrix structural constituents, in the GO_biological_process (Figure 7C), GO_cellular_component (Supplementary Figure S5A), and GO_molecular_function (Supplementary Figure S5B) categories. Then, we displayed the extracellular matrix-associated terms in GOCircle (Figure 7D) and chord (Figure 7E) plots.

KEGG analysis data identified the ECM-receptor interaction (Supplementary Figure S6). GSEA data also showed the extracellular matrix (ECM)-associated gene sets, including extracellular structure organization, extracellular matrix structural constituents, ECM-receptor interaction, miRNA targets in ECM and membrane receptors (Supplementary Figure S7). Based on the GSCALite pathway score analysis, we further observed activation of the epithelial-mesenchymal transition (EMT) pathway (Supplementary Figure S8).

### PCA and Random Forest Analysis Data

**FIGURE 8:**
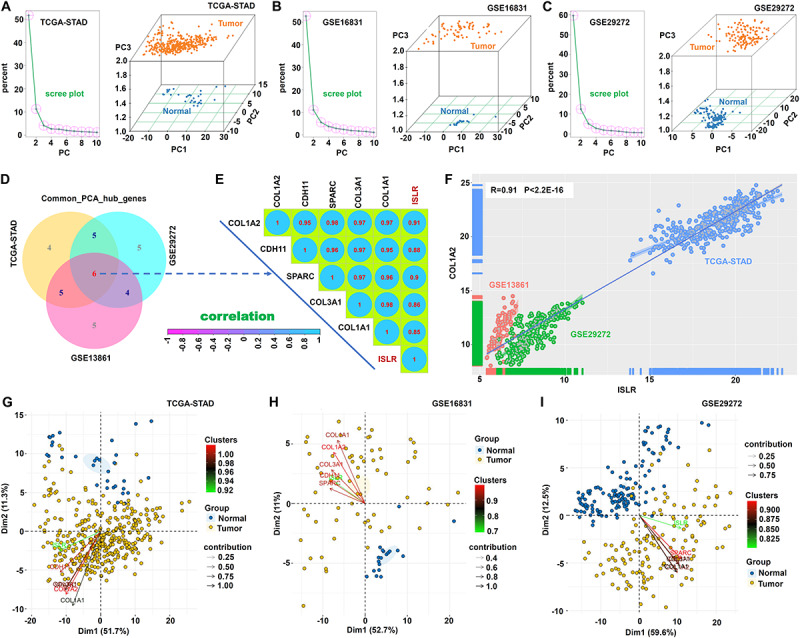
Principal component analysis. Based on the common_POS_genes and common_NEG_genes, the prcomp function was used for PCA to classify the normal and tumor sections in TCGA-STAD **(A)**, GSE13861 **(B)**, and GSE29272 **(C)**. A scree plot and a three-dimensional map (PC1, PC2, and PC3) are provided. **(D)** The “VennDiagram” R package was used for the common_PCA_hub_genes. **(E)** The cor function and “corrplot” R package were used for the Spearman correlation analysis of these hub genes. Correlation coefficients are shown. **(F)** The scatter plot for the correlation between *ISLR* and *COL1A2* gene expression is provided. **(G–I)** The “factoextra” R package was utilized to show the principal component weight and two-dimensional contribution maps of common hub genes.

To further identify *ISLR*-correlated hub genes for the differentiation of tumor from normal samples, we performed PCA. As shown in Figures 8A–C, we used PC1, PC2 and PC3 to distinguish normal from tumor samples in the three datasets: TCGA-STAD, GSE13861, and GSE29272. Then, we conducted an intersection analysis and identified six hub genes, *ISLR*, *COL1A2* (collagen type I alpha 2 chain), *CDH11* (cadherin 11), *SPARC* (secreted protein acidic and cysteine-rich), *COL3A1* (collagen type III alpha 1 chain), and *COL1A1* (collagen type I alpha 1 chain) (Figure 8D). There were strong positive correlations of expression among these genes, and all correlation coefficients were greater than 0.8 (Figure 8E). Figure 8F presents the correlation between *ISLR* and *COL1A2* gene expression in the three datasets (*R* = 0.91, *P* < 2.2E-16). Figures 8G–I further shows the contribution of these hub genes to PC1 and PC2.

**FIGURE 9:**
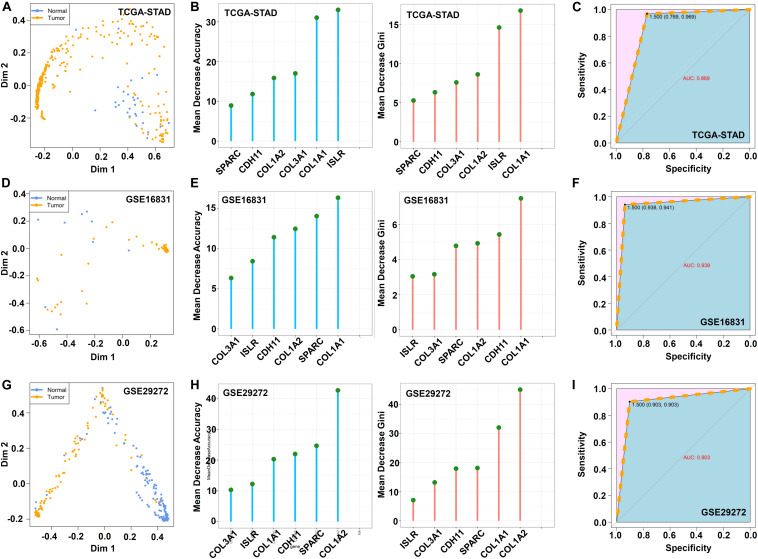
Random forest analysis. Based on the above hub genes, the “randomForest” package (ntree = 500) was utilized for the random forest modeling analysis. The MDSplot function was used to obtain a multidimensional scale **(A,D,G)**. The data of mean decrease accuracy and mean decrease Gini were visualized by the ggdotchart function in the “ggpubr” package **(B,E,H)**. The receiver operating characteristic (ROC) curve was plotted by the “pROC” package, and the area under the ROC curve (AUC) value was calculated **(C,F,I)**.

Subsequently, we carried out a random forest analysis based on these six hub genes. The multidimensional scale plot in Figure 9A suggests the effective differentiation of normal from tumor samples in the TCGA-STAD cohort. Figure 9B shows the mean decrease accuracy and mean decrease Gini data. The AUC value of 0.869 indicated high classification accuracy (Figure 9C). Similar results were observed in the GSE16831 (Figures 9D–F) and GSE29272 (Figures 9G–I) datasets.

### Drug Sensitivity Analysis Data

Finally, based on the CTRP database, we conducted a small molecule/drug sensitivity (IC50) evaluation and further detected that the expression of *the ISLR* gene was negatively related to sensitivity to PX-12 and NSC632839 (Supplementary Figure S9).

## Discussion

Based on the available datasets of gastric cancer cases collected by TCGA and GEO, for the first time, we found a statistical correlation between high expression of the *ISLR* gene and poor overall survival, disease-free survival, first progression, and post-progression survival. There were significant differences in *ISLR* expression among different pathological stages (stages 1–4). When gastric cancer samples were divided by clinical information, a positive correlation between *ISLR* expression and gastric cancer prognosis existed in most subgroups, such as subgroups based on different Lauren classifications (intestinal or diffuse). Notably, we only observed a correlation between *ISLR* gene expression and OS in the well-differentiated subgroup but not in the poorly or moderately differentiated subgroup. In addition, we detected a positive effect of *ISLR* expression on survival in the pathological stage 3 subgroup but not the stage 1, 2, or 4 subgroups. These results implied that the prognostic ability of high *ISLR* gene expression may increase with tumor differentiation or pathological grade.

Upon integrated analysis, we observed that high expression of the *ISLR* gene showed a correlation with low sensitivity to PX-12 (an irreversible inhibitor of thioredoxin-1) (Metcalfe et al., 2016) and NSC632839 (a non-selective isopeptidase inhibitor) (Nicholson et al., 2008), indicating that high *ISLR* gene expression may be associated with chemoresistance in gastric cancer. Unexpectedly, during the survival analysis of gastric cancer patients treated with a 5-Fu-based adjuvant, high expression of the *ISLR* gene was linked to a better prognosis than low expression of the *ISLR* gene. It is possible that 5-Fu treatment interferes with the expression of the *ISLR* gene in patients, which leads to changes in survival outcomes. Additionally, although we did not detect a correlation between *ISLR* gene expression and 5-Fu drug sensitivity through our preliminary assessment, more clinical gastric cancer samples specifically under the treatment of 5-Fu and comprehensive analysis are needed to validate the relationship between *ISLR* expression and 5-Fu chemotherapy resistance.

DNA methylation status is closely associated with the carcinogenesis or drug resistance of gastric cancer (Tahara and Arisawa, 2015; Choi et al., 2017). Although we failed to detect a correlation between *ISLR* gene mutations or CNVs and the clinical prognosis of gastric cancer, the hypomethylation status of several sites within *ISLR* (cg05195566, cg17258195, cg09664357, and cg0729703) was linked to high expression of *ISLR* and clinically poor survival outcomes. We noted that the cg05195566 and cg17258195 sites are situated in the promoter region, while cg09664357 and cg0729703 are outside the promoter region. It is worthwhile to further investigate how methylation of different sites within *ISLR* affects the expression level and survival outcomes of gastric cancer patients.

Considering the structure of the ISLR protein as a member of the Ig superfamily (Nagasawa et al., 1997) and the functional links between immune infiltration and gastric cancer (Kim et al., 2016; Liu et al., 2016; Pan et al., 2019; Xu et al., 2019), we first investigated the correlation between *ISLR* gene expression and macrophage, neutrophil, dendritic cell, B cell, T cell and other immune cell infiltration levels based on gene expression correlations and the TIMER, CIBERSORT, CIBERSORT-ABS, QUANTISEQ, MCPCOUNTER, XCELL, and EPIC algorithms. The results were adjusted for tumor purity. We observed a positive correlation between *ISLR* gene expression and several immune cells, especially CD8^+^ T cells, macrophages and dendritic cells. We also detected a correlation between *ISLR* CNV and immune infiltration. These results indicated that the tumor microenvironment may be key in the complex molecular mechanism by which *the ISLR* gene affects carcinogenesis of gastric cancer.

The extracellular matrix (ECM) and epithelial-mesenchymal transition (EMT) have been reported to be associated with the invasion and migration of gastric cancer (Lukaszewicz-Zajac et al., 2011; Peng et al., 2014; Huang et al., 2015). After pooling the *ISLR* expression-associated genes, we detected significantly enriched ECM-related pathways, including miRNA targets in the ECM and membrane receptors. Our PCA and random forest analysis further identified six extracellular matrix-associated hub genes, which were able to distinguish between gastric cancer and normal control samples. We also found that *ISLR* gene expression was associated with the activation of the EMT pathway. Considering the connection between miRNAs and EMT in gastric cancer (Bure et al., 2019), we performed GSCALite mRNA-miRNA regulation network analysis to identify several potential *ISLR*-binding miRNAs, including hsa-miR16-5p, hsa-miR-3116, hsa-mir-934, hsa-miR98-5p, and hsa-miR-339-5p (data not shown). It is meaningful to evaluate the relationship between *ISLR* expression and EMT from the perspective of miRNA and to investigate the mechanism underlying the progression of gastric cancer. In addition, the chief aim of our research was only to examine the potential mechanism by which the *ISLR* gene participates in gastric carcinogenesis. It should be noted that *ISLR* does not show specificity for gastric cancer tissue (data not shown), and its role in other cancers cannot be ruled out. Most likely, ISLR works as an effective prognostic marker during gastric carcinogenesis because it forms functional protein-protein and protein-nucleic acid complexes.

## Conclusion

After our bioinformatics and biostatistics analyses of gastric cancer cases within the TCGA and GEO cohorts, high *ISLR* expression was identified as a potential prognostic biomarker for gastric cancer. DNA hypomethylation of *ISLR* is linked to high expression of the *ISLR* gene and overall clinical prognosis. *ISLR* expression was also correlated with the infiltration of several immune cells (e.g., CD8^+^ T cells, macrophages and dendritic cells), EMT pathway activity and sensitivity to PX-12 and NSC632839. Our findings are of great significance for conducting ISLR-based cell or animal experimental validation.

## Data Availability Statement

The datasets generated for this study are available on request to the corresponding author.

## Author Contributions

SL conceived and designed the study and drafted the manuscript. SL and WZ performed the *ISLR* expression, survival curve analysis, and genetic alteration analysis. SL and MS performed the DNA methylation, immune cell infiltration analysis, *ISLR*-correlated pathway, PCA, random forest, and drug sensitivity analysis. All authors reviewed the manuscript before submission and approved the final version of the manuscript.

## Conflict of Interest

WZ was employed by the General Data Technology Co., Ltd, Tianjin. The remaining authors declare that the research was conducted in the absence of any commercial or financial relationships that could be construed as a potential conflict of interest.

## Footnotes

1 http://gepia2.cancer-pku.cn/#analysis

2 https://www.oncomine.org/resource/main.html

3 https://www.r-project.org/

4 https://www.cbioportal.org/

5 https://xenabrowser.net/

## Supplementary Material

FIGURE S1 Correlation between methylation status and expression of *ISLR* in gastric cases in the TCGA-STAD cohort. Detailed information on the methylation probe is provided. Pearson correlation coefficients (R) and Benjamini–Hochberg-adjusted *P*-values (^∗^*P* < 0.05, ^∗∗∗^*P* < 0.001) for the comparison are shown as well.

FIGURE S2 Correlation between the methylation status of *ISLR* DNA and the survival rate of gastric patients in the TCGA-STAD cohort. A total of eleven methylation probes, including cg15480336 **(A)**, cg03778780 **(B)**, cg02077702 **(C)**, cg20536146 **(D)**, cg11335960 **(E)**, cg24779381 **(F)**, cg05195566 **(G)**, cg177258195 **(H)**, cg09664357 **(I)**, cg07297039 **(J)**, and cg16926502 **(K)**, were used.

FIGURE S3 Correlation between *ISLR* and markers of T or B cells as analyzed through GEPIA2. **(A)** B cells; **(B)** Th1 cells; **(C)** Th2 cells; **(D)** Th17 cells; **(E)** CD8^+^ T cells; **(F)** Tfh cells; **(G)** resting Treg cells; **(H)** effector Treg cells; **(I)** exhausted T cells.

FIGURE S4 Correlation between *ISLR* CNV and the infiltration level of immune cells as analyzed through TIMER. **(A)** dendritic cells; **(B)** neutrophils; **(C)** CD8^+^ T cells; **(D)** CD4^+^ T cells; **(E)** B cells; **(F)** macrophages. (^∗^*P* < 0.05, ^∗∗^*P* < 0.01, ^∗∗∗^*P* < 0.001).

FIGURE S5 GO_biological_process and GO_molecular_function enrichment analysis. The “clusterProfiler” R package was used for the GO_biological_process **(A)** and GO_molecular_function **(B)** enrichment analyses.

FIGURE S6 KEGG enrichment analysis. The “pathview” R package was used for the KEGG enrichment analysis.

FIGURE S7 GSEA. A LinkedOmics approach was utilized for the *ISLR*-correlated GSEA profiles. Four extracellular matrix (ECM)-associated gene sets, including genes involved in extracellular structural organization **(A)**, extracellular matrix structural constituents **(B)**, ECM-receptor interactions **(C)**, and miRNA targets in the ECM and membrane receptors **(D)**, were identified.

FIGURE S8 Correlation between *ISLR* expression and pathway activation or inhibition.

FIGURE S9 Correlation between *ISLR* expression and small molecule/drug sensitivity (IC50).

TABLE S1 Correlation of *ISLR* expression and the first progression status of gastric cancer patients in the GEO cohort (Kaplan–Meier plotter).

TABLE S2 Correlation of *ISLR* expression and the PPS of gastric cancer patients in the GEO cohort (Kaplan–Meier plotter).
