# Supplementary material for: An Analysis Regarding the Association Between the ISLR Gene and Gastric Carcinogenesis
Source: Front Genet. 2020 Jun 16;11:620. doi: 10.3389/fgene.2020.00620 (PMC7308588; doi:10.3389/fgene.2020.00620)
Supplement: FIGURE S1 — Correlation between methylation status and expression of ISLR in gastric cases in the TCGA-STAD cohort. Detailed information on the methylation probe is provided. Pearson correlation coefficients (R) and Benjamini–Hochberg-adjusted P-values (∗P < 0.05, ∗∗∗P < 0.001) for the comparison are shown as well. [file Data_Sheet_1.pdf]

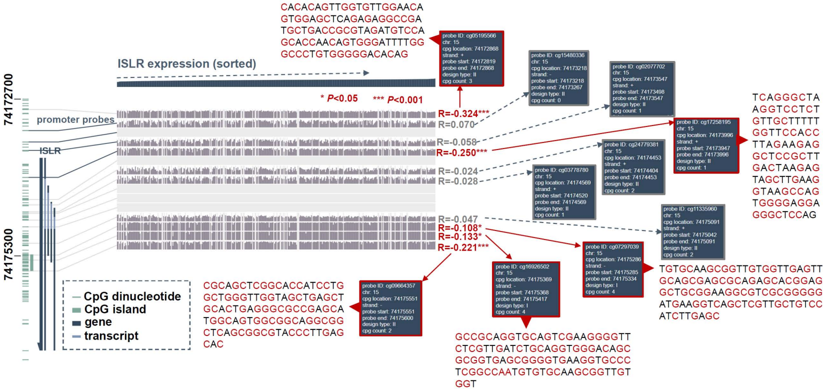

CACACAGTTGGTGTTGGAACA
GTGGAGCTCAGAGAGGCCGA
TGCTGACCGCGTAGATGTCCA
GCACCAACAGTGGGATTTTGG
GCCCTGTGGGGGACACAG
ISLR expression (sorted)
74172700
74175300
promoter probes
ISLR
* P<0.05
*** P<0.001
R=-0.324***
R=0.070
R=-0.058
R=-0.250***
R=-0.024
R=-0.028
R=-0.047
R=-0.108*
R=-0.133*
R=-0.221***
probe ID: cg05195566 chr: 15 cpg location: 74172868 strand: + probe start: 74172819 probe end: 74172868 design type: II cpg count: 3
probe ID: cg15480336 chr: 15 cpg location: 74173218 strand: - probe start: 74173218 probe end: 74173267 design type: II cpg count: 0
probe ID: cg02077702 chr: 15 cpg location: 74173547 strand: + probe start: 74173498 probe end: 74173547 design type: II cpg count: 1
probe ID: cg17258195 chr: 15 cpg location: 74173996 strand: + probe start: 74173947 probe end: 74173996 design type: II cpg count: 1
probe ID: cg24779381 chr: 15 cpg location: 74174453 strand: + probe start: 74174404 probe end: 74174453 design type: II cpg count: 2
probe ID: cg03778780 chr: 15 cpg location: 74174569 strand: + probe start: 74174520 probe end: 74174569 design type: II cpg count: 1
probe ID: cg11335960 chr: 15 cpg location: 74175091 strand: + probe start: 74175042 probe end: 74175091 design type: II cpg count: 2
probe ID: cg07297039 chr: 15 cpg location: 74175286 strand: - probe start: 74175285 probe end: 74175334 design type: I cpg count: 4
probe ID: cg16926502 chr: 15 cpg location: 74175369 strand: - probe start: 74175368 probe end: 74175417 design type: I cpg count: 4
probe ID: cg09664357 chr: 15 cpg location: 74175551 strand: - probe start: 74175551 probe end: 74175600 design type: II cpg count: 2
TCAGGGCTA
AGGTCCTCT
GTTGCTTTTT
GGTTCCACC
TTAGAAGAG
GCTCCGCTT
GACTAAGAG
TAGCTTGAAG
GTAAGCCAG
TGGGGAGGA
GGGCTCCAG
TGTGCAAGCGGTTGTGGTTGAGTT
GCAGCGAGCGCAGAGCACGGAG
GCTGCGGAAGGCGTCGCGGGGG
ATGAAGGTCAGCTCGTTGCTGTCC
ATCTTGAGC
GCCGCAGGTGCAGTCGAAGGGGTT
CTCGTTGATCTGCAGGTGGGACAGC
GCGGTGAGCGGGGTGAAGGTGCCC
TCGGCCAATGTGTGCAAGCGGTTGT
GGT
CGCAGCTCGGCACCATCCTG
GCTGGGTTGGTAGCTGAGCT
GCACTGAGGGCGCCGAGCA
TGGCAGTGGCGGCAGGCGG
CTCAGCGGCGTACCCTTGAG
CAC
CpG dinucleotide
CpG island
gene
transcript

Figure S1

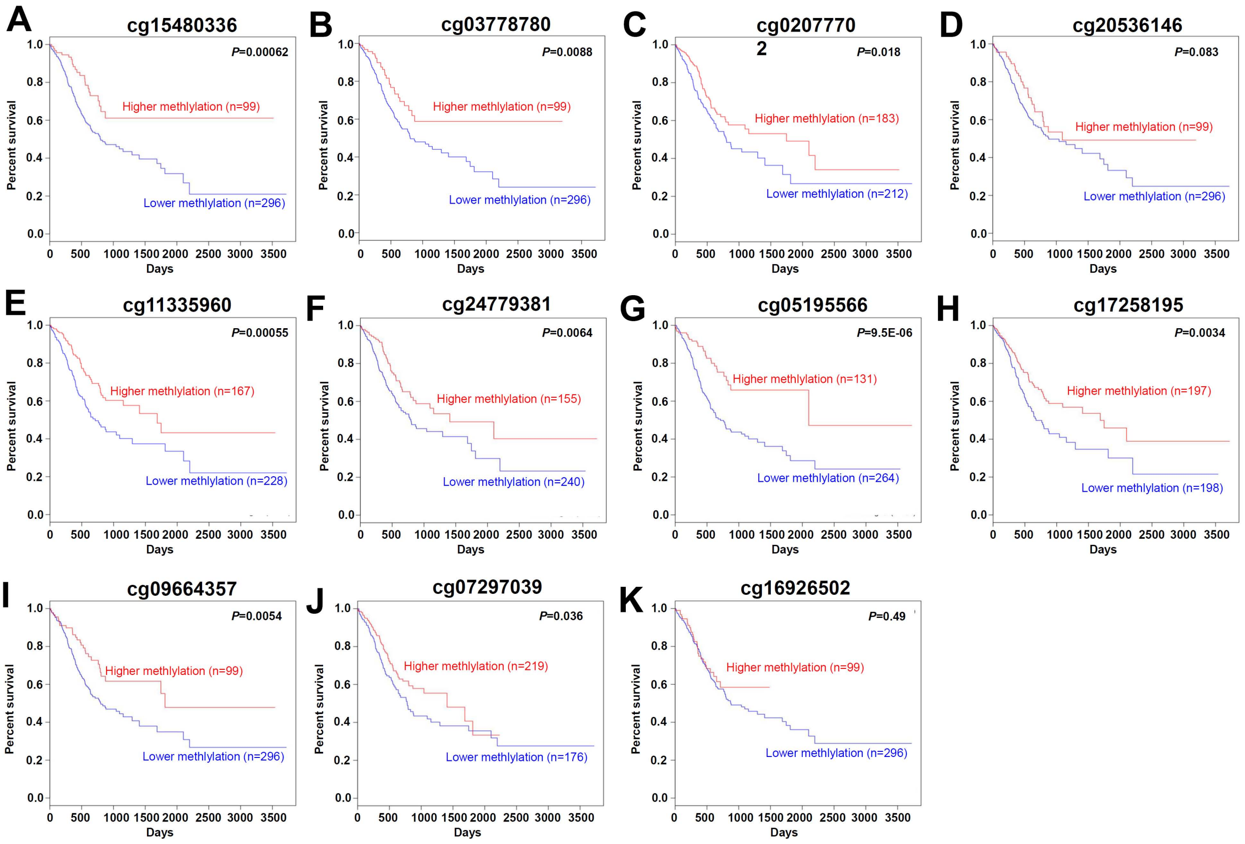
A
cg15480336
P=0.00062
Higher methlylation (n=99)
Lower methlylation (n=296)
B
cg03778780
P=0.0088
Higher methlylation (n=99)
Lower methlylation (n=296)
C
cg0207770
2
P=0.018
Higher methlylation (n=183)
Lower methlylation (n=212)
D
cg20536146
P=0.083
Higher methlylation (n=99)
Lower methlylation (n=296)
E
cg11335960
P=0.00055
Higher methlylation (n=167)
Lower methlylation (n=228)
F
cg24779381
P=0.0064
Higher methlylation (n=155)
Lower methlylation (n=240)
G
cg05195566
P=9.5E-06
Higher methlylation (n=131)
Lower methlylation (n=264)
H
cg17258195
P=0.0034
Higher methlylation (n=197)
Lower methlylation (n=198)
I
cg09664357
P=0.0054
Higher methlylation (n=99)
Lower methlylation (n=296)
J
cg07297039
P=0.036
Higher methlylation (n=219)
Lower methlylation (n=176)
K
cg16926502
P=0.49
Higher methlylation (n=99)
Lower methlylation (n=296)
Percent survival
Days
0.0
0.2
0.4
0.6
0.8
1.0
0
500
1000
1500
2000
2500
3000
3500

Figure S2

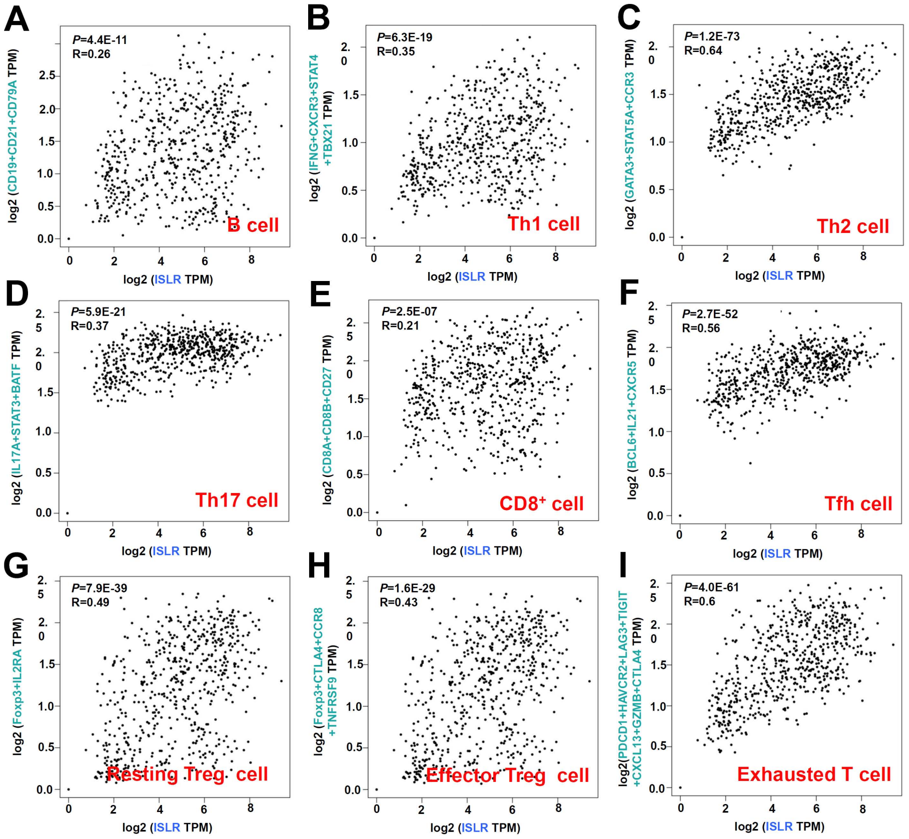

A
P=4.4E-11
R=0.26
B cell
log2 (CD19+CD21+CD79A TPM)
log2 (ISLR TPM)
B
P=6.3E-19
R=0.35
Th1 cell
log2 (IFNG+CXCR3+STAT4+TBX21 TPM)
log2 (ISLR TPM)
C
P=1.2E-73
R=0.64
Th2 cell
log2 (GATA3+STAT5A+CCR3 TPM)
log2 (ISLR TPM)
D
P=5.9E-21
R=0.37
Th17 cell
log2 (IL17A+STAT3+BATF TPM)
log2 (ISLR TPM)
E
P=2.5E-07
R=0.21
CD8+ cell
log2 (CD8A+CD8B+CD27 TPM)
log2 (ISLR TPM)
F
P=2.7E-52
R=0.56
Tfh cell
log2 (BCL6+IL21+CXCR5 TPM)
log2 (ISLR TPM)
G
P=7.9E-39
R=0.49
Resting Treg cell
log2 (Foxp3+IL2RA TPM)
log2 (ISLR TPM)
H
P=1.6E-29
R=0.43
Effector Treg cell
log2 (Foxp3+CTLA4+CCR8+TNFRSF9 TPM)
log2 (ISLR TPM)
I
P=4.0E-61
R=0.6
Exhausted T cell
log2(PDCD1+HAVCR2+LAG3+TIGIT+CXCL13+GZMB+CTLA4 TPM)
log2 (ISLR TPM)

Figure S3

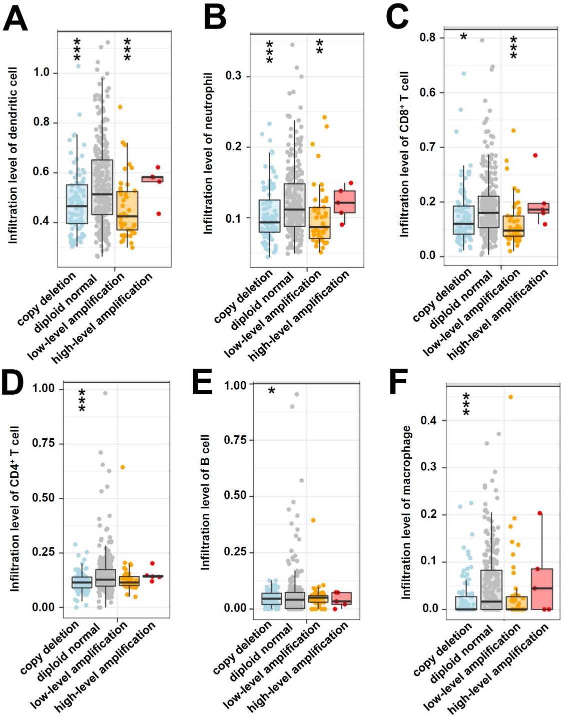
A
B
C
D
E
F
Infiltration level of dendritic cell
Infiltration level of neutrophil
Infiltration level of CD8+ T cell
Infiltration level of CD4+ T cell
Infiltration level of B cell
Infiltration level of macrophage
copy deletion
diploid normal
low-level amplification
high-level amplification

Figure S4

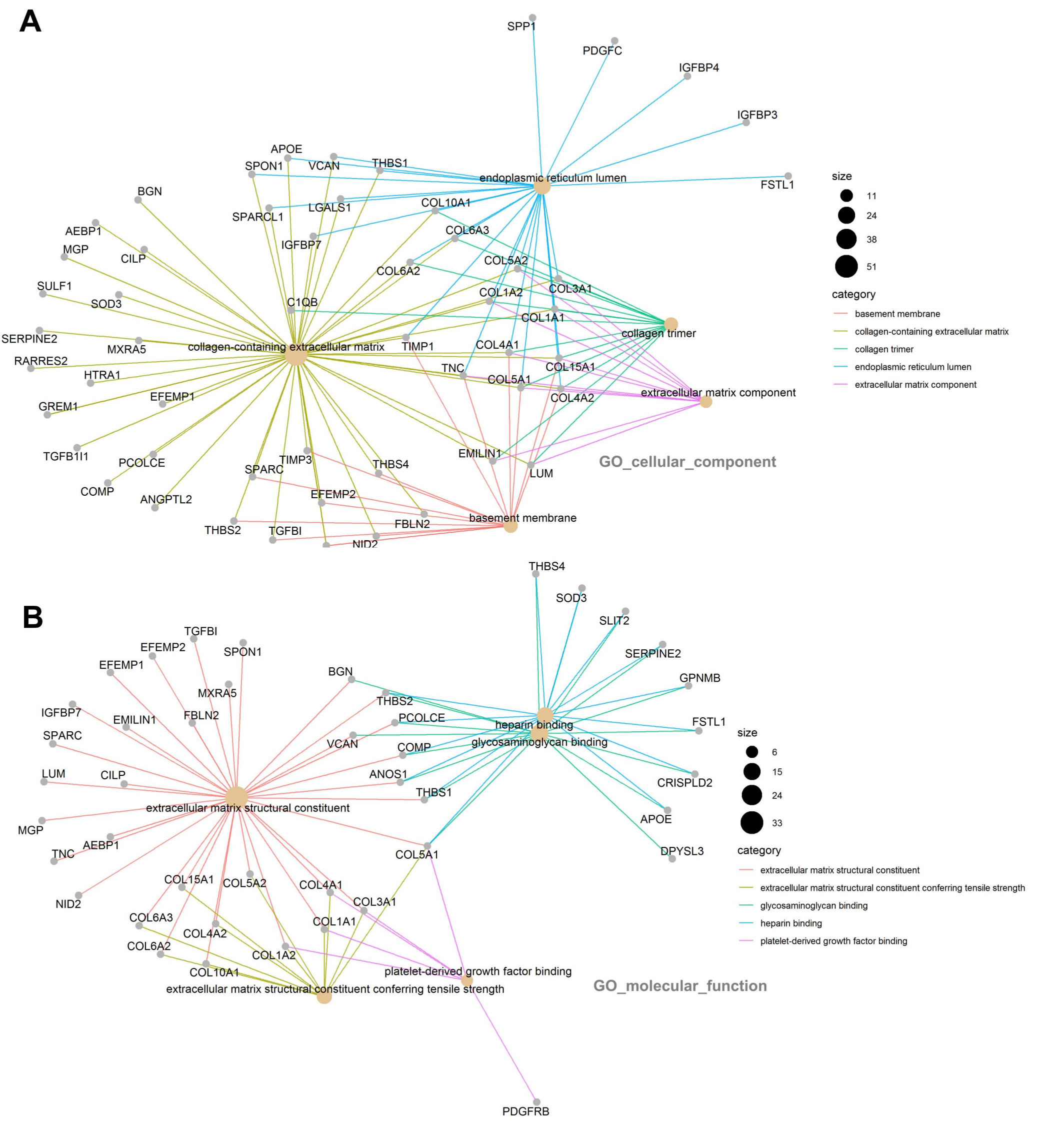
A
SPP1
PDGFC
IGFBP4
IGFBP3
FSTL1
endoplasmic reticulum lumen
APOE
SPON1
VCAN
THBS1
BGN
LGALS1
SPARCL1
COL10A1
AEBP1
MGP
CILP
IGFBP7
COL6A3
COL6A2
COL5A2
SULF1
SOD3
C1QB
COL1A2
COL3A1
COL1A1
collagen trimer
SERPINE2
MXRA5
collagen-containing extracellular matrix
TIMP1
COL4A1
RARRES2
HTRA1
TNC
COL15A1
COL5A1
GREM1
EFEMP1
COL4A2
extracellular matrix component
TGFB1I1
PCOLCE
COMP
ANGPTL2
SPARC
TIMP3
THBS4
EMILIN1
LUM
GO_cellular_component
EFEMP2
THBS2
TGFBI
FBLN2
NID2
basement membrane
size
11
24
38
51
category
basement membrane
collagen-containing extracellular matrix
collagen trimer
endoplasmic reticulum lumen
extracellular matrix component
B
THBS4
SOD3
SLIT2
SERPINE2
GPNMB
FSTL1
TGFBI
EFEMP2
SPON1
EFEMP1
MXRA5
BGN
IGFBP7
EMILIN1
FBLN2
THBS2
PCOLCE
heparin binding
glycosaminoglycan binding
SPARC
VCAN
COMP
LUM
CILP
ANOS1
CRISPLD2
THBS1
extracellular matrix structural constituent
APOE
MGP
TNC
AEBP1
COL5A1
DPYSL3
NID2
COL15A1
COL5A2
COL4A1
COL3A1
COL6A3
COL1A1
COL4A2
COL6A2
COL1A2
COL10A1
platelet-derived growth factor binding
extracellular matrix structural constituent conferring tensile strength
GO_molecular_function
PDGFRB
size
6
15
24
33
category
extracellular matrix structural constituent
extracellular matrix structural constituent conferring tensile strength
glycosaminoglycan binding
heparin binding
platelet-derived growth factor binding

Figure S5

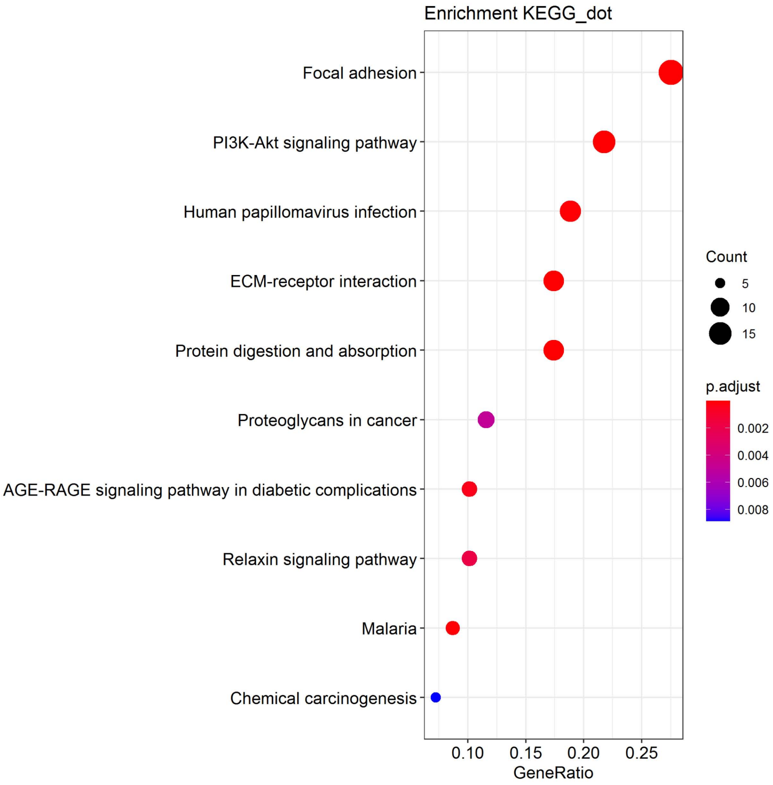
Enrichment KEGG_dot
Focal adhesion
PI3K-Akt signaling pathway
Human papillomavirus infection
ECM-receptor interaction
Protein digestion and absorption
Proteoglycans in cancer
AGE-RAGE signaling pathway in diabetic complications
Relaxin signaling pathway
Malaria
Chemical carcinogenesis
0.10
0.15
0.20
0.25
GeneRatio
Count
5
10
15
p.adjust
0.002
0.004
0.006
0.008

Figure S6

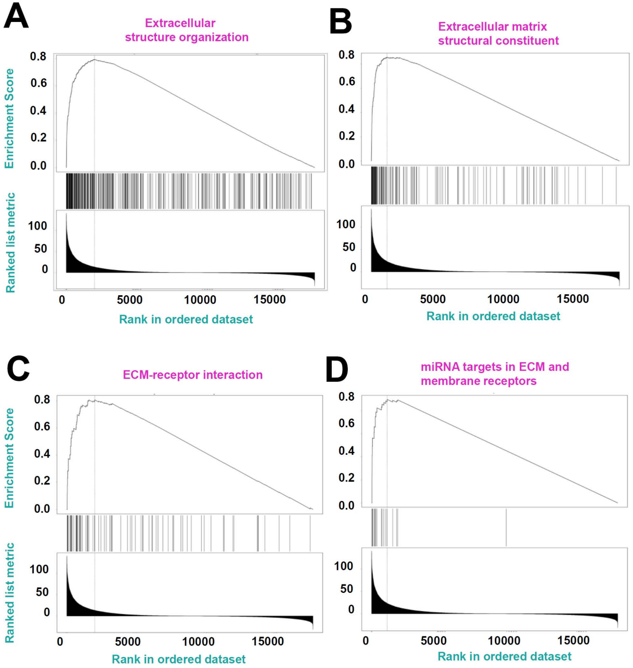
A
Extracellular structure organization
B
Extracellular matrix structural constituent
C
ECM-receptor interaction
D
miRNA targets in ECM and membrane receptors
Enrichment Score
0.8
0.6
0.4
0.2
0.0
Ranked list metric
100
50
0
0
5000
10000
15000
Rank in ordered dataset

Figure S7

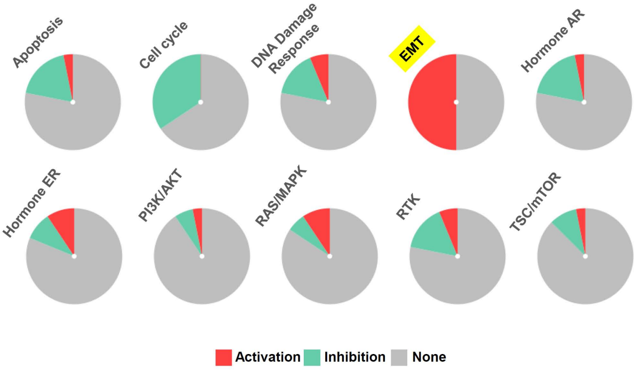

Apoptosis
Cell cycle
DNA Damage Response
EMT
Hormone AR
Hormone ER
PI3K/AKT
RAS/MAPK
RTK
TSC/mTOR
Activation
Inhibition
None

Figure S8

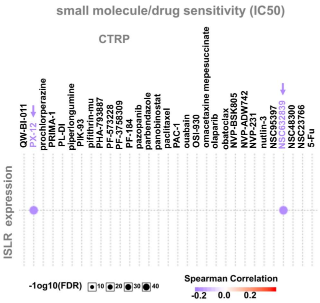
small molecule/drug sensitivity (IC50)
CTRP
QW-BI-011
PX-12
prochlorperazine
PRIMA-1
PL-DI
piperlongumine
PIK-93
pifithrin-mu
PHA-793887
PF-573228
PF-3758309
PF-184
pazopanib
parbendazole
panobinostat
paclitaxel
PAC-1
ouabain
OSI-930
omacetaxine mepesuccinate
olaparib
obatoclax
NVP-BSK805
NVP-ADW742
NVP-231
nutlin-3
NSC95397
NSC632839
NSC48300
NSC23766
5-Fu
ISLR expression
-1og10(FDR)
10
20
30
40
Spearman Correlation
-0.2
0.0
0.2

Figure S9
