# Supplementary material for: An Analysis Regarding the Association Between the ISLR Gene and Gastric Carcinogenesis
Source: Front Genet. 2020 Jun 16;11:620. doi: 10.3389/fgene.2020.00620 (PMC7308588; doi:10.3389/fgene.2020.00620)
Supplement: TABLE S1 — Correlation of ISLR expression and the first progression status of gastric cancer patients in the GEO cohort (Kaplan–Meier plotter). [file Data_Sheet_2.docx]

## TABLE S1: Correlation of *ISLR* expression and the first progression status of gastric cancer patients in the GEO cohort (Kaplan-Meier plotter)

| **Factor** | **Group** | **Sample size** | **HR** | **95% CI** | ***logRank_P*** |
| --- | --- | --- | --- | --- | --- |
| **Gender** | Female | 244 | 2.2 | 1.46-3.33 | **0.00013** |
|  | Male | 567 | 1.72 | 1.34-2.22 | **1.7E-05** |
| **Stage** | Stage 1 | 69 | 1.76 | 0.54-5.76 | **0.35** |
|  | Stage 2 | 145 | 1.63 | 0.88-3.03 | **0.12** |
|  | Stage 3 | 319 | 2.45 | 1.56-3.86 | **6.3E-05** |
|  | Stage 4 | 152 | 1.45 | 0.98-2.16 | **0.063** |
| **Stage T** | T2 | 253 | 1.64 | 1.07-2.53 | **0.023** |
|  | T3 | 208 | 1.65 | 1.13-2.42 | **0.0093** |
|  | T4 | 39 | 2.52 | 1.1-5.79 | **0.025** |
| **Stage N** | N0 | 76 | 2.84 | 1.1-7.36 | **0.025** |
|  | N1 | 232 | 2.51 | 1.65-3.81 | **7.7E-06** |
|  | N2 | 129 | 2.33 | 1.44-3.77 | **0.00041** |
|  | N3 | 76 | 1.9 | 1.09-3.32 | **0.022** |
|  | N1+2+3 | 437 | 1.95 | 1.51-2.53 | **2.7E-07** |
| **Stage M** | M0 | 459 | 1.85 | 1.41-2.43 | **7.5E-06** |
|  | M1 | 58 | 1.62 | 0.87-2.99 | **0.12** |
| **HER2** | negative | 641 | 1.84 | 1.4-2.42 | **9.2E-06** |
|  | positive | 425 | 1.7 | 1.19-2.43 | **0.0033** |
| **Lauren Classification** | Instestinal | 336 | 2.37 | 1.62-3.48 | **5.3E-06** |
|  | Diffuse | 248 | 1.75 | 1.23-2.50 | **0.0016** |
| **Differentiation** | Poorly | 166 | 1.65 | 1.01-2.69 | **0.044** |
|  | Moderately | 67 | 1.9 | 1.02-3.56 | **0.04** |
| **Treatment** | Surgery alone | 393 | 1.59 | 1.1-2.3 | **0.014** |
|  | 5-Fu based adjuvant | 158 | 0.66 | 0.46-0.95 | **0.023** |
|  | Other adjuvant | 80 | 2.5 | 1.13-5.5 | **0.019** |

*HR: hazard ratio; CI: confidence interval; HER2: Erb-B2 Receptor Tyrosine Kinase 2; logRank_P* value less than 0.05 was shown in bold.

## TABLE S2: Correlation of *ISLR* expression and the PPS of gastric cancer patients in the GEO cohort (Kaplan-Meier plotter)

| **Factor** | **Group** | **Sample size** | **HR** | **95% CI** | ***logRank_P*** |
| --- | --- | --- | --- | --- | --- |
| **Gender** | Female | 244 | 3.36 | 1.97-5.72 | **2.2E-06** |
|  | Male | 567 | 2.6 | 2-3.38 | **1.8E-13** |
| **Stage** | Stage 1 | 69 | 4.78 | 0.97-23.58 | **0.04** |
|  | Stage 2 | 145 | 2.28 | 1.18-4.44 | **0.012** |
|  | Stage 3 | 319 | 3.62 | 2.22-5.9 | **4.0E-08** |
|  | Stage 4 | 152 | 1.84 | 1.16-2.93 | **0.0092** |
| **Stage T** | T2 | 253 | 2.28 | 1.45-3.58 | **0.00023** |
|  | T3 | 208 | 2.68 | 1.7-4.22 | **1E-05** |
|  | T4 | 39 | 1.91 | 0.55-6.65 | 0.3 |
| **Stage N** | N0 | 76 | 5.41 | 1.61-18.22 | **0.0023** |
|  | N1 | 232 | 3.39 | 2.14-5.38 | **4.1E-08** |
|  | N2 | 129 | 3.34 | 1.95-5.71 | **3.2E-06** |
|  | N3 | 76 | 1.8 | 1-3.23 | **0.045** |
|  | N1+2+3 | 437 | 2.68 | 2.01-3.58 | **3.4E-12** |
| **Stage M** | M0 | 459 | 3 | 2.22-4.06 | **8.9E-14** |
|  | M1 | 58 | 3.19 | 3.51-7.51 | **0.0055** |
| **HER2** | Negative | 641 | 2.4 | 1.8-3.2 | **7.4E-10** |
|  | Positive | 425 | 2.57 | 1.78-3.7 | **1.9E-07** |
| **Lauren Classification** | Instestinal | 336 | 3.51 | 2.31-5.33 | **3.8E-10** |
|  | Diffuse | 248 | 2.45 | 1.66-3.64 | **4E-06** |
| **Differentiation** | Poor | 166 | 1.66 | 0.86-3.2 | 0.13 |
|  | Moderate | 67 | 1.92 | 0.77-4.78 | 0.16 |
| **Treatment** | Surgery alone | 393 | 2.46 | 1.8-3.37 | **6.8E-09** |
|  | 5-Fu based adjuvant | 158 | 0.65 | 0.44-0.97 | **0.034** |
|  | Other adjuvant | 80 | 2.59 | 1.07-6.24 | **0.028** |

*HR: hazard ratio; CI: confidence interval; HER2: Erb-B2 Receptor Tyrosine Kinase 2;*

*logRank_P* value less than 0.05 was shown in bold.
